# Can Telehealth Improve Access to Dietary Management in Patients Receiving Dialysis? Insights from Consumers

**DOI:** 10.3390/nu16010105

**Published:** 2023-12-28

**Authors:** Joanne Beer, Kelly Lambert, Wai Lim, Catherine Keane, Neil Boudville

**Affiliations:** 1 Nutrition and Dietetics Department, Sir Charles Gairdner Hospital, Nedlands, WA 6009, Australia; 2 School of Medical, Indigenous and Health Sciences, Faculty of Science, Medicine and Health, University of Wollongong, Wollongong, NSW 2522, Australia; klambert@uow.edu.au; 3 Department of Renal Medicine, Sir Charles Gairdner Hospital, Nedlands, WA 6009, Australia; wai.lim@health.wa.gov.au (W.L.); neil.boudville@uwa.edu.au (N.B.); 4 Independent Researcher, Floreat, WA 6014, Australia; 5 Medical School, University of Western Australia, Crawley, WA 6009, Australia

**Keywords:** consumer perspectives, dialysis, dietary education, kidney failure, telehealth, qualitative

## Abstract

Timely, effective, and individualised dietary interventions are essential for patients undergoing dialysis. However, delivery of dietary advice is challenging due to limited access to renal dietitians, as well as logistic and scheduling difficulties for patients receiving dialysis. The objectives of this study were to explore consumer perspectives regarding dietary advice utilising telehealth technology. Twenty-two participants (seventeen patients receiving dialysis, five caregivers) were purposively recruited from a local dialysis centre and participated in one of three focus groups. Each focus group was recorded, transcribed, and analysed using inductive thematic analysis. One overarching theme: “a desire to learn” was apparent. The four themes that facilitated this process are herein described: Meaningful *communication*—a need for improved and individualised communication about diet using positively framed messages with consistency among clinicians. Conducive *information*—a preference for tailored, current, and clear dietary information (plain language was preferred, with practical advice on making dietary changes). *Appropriate timing*—health advice at the right time (consumers felt overwhelmed, not supported enough with timely advice, and experienced difficulty attending appointments in addition to dialysis treatments). *Contemporary modalities*—delivering information using different technologies (consumers preferred a combination of delivery methods for dietetic advice including text/SMS/App messages as an adjunct to face-to-face care). The results showed that consumers believe that telehealth options are an acceptable adjunct to receive dietary advice in a timely manner, and feedback from patients and caregivers has informed the design of a clinical trial to incorporate the use of telehealth to improve the management of serum phosphate.

## 1. Introduction

Hyperphosphatemia in patients with kidney failure is common, with reports indicating that over half of patients on haemodialysis have pre-dialysis hyperphosphatemia (serum phosphate levels of >1.78 mmol/L) [1,2]. Recently, Yin et al. [3] reported a considerably higher prevalence of 73% among those receiving continuous ambulatory peritoneal dialysis.

Hyperphosphatemia is associated with increased morbidity and mortality [4], which may be due to an increased risk of vascular calcification, coronary artery disease, heart failure, metabolic bone disease (renal osteodystrophy), and the development of secondary hyperparathyroidism [5]. 

Reduction in serum phosphate levels can be achieved through more efficient dialysis, reduced dietary phosphate intake, and the administration of phosphate binding medications. However, management of hyperphosphatemia remains a major challenge for patients [6] and healthcare professionals due to poor adherence to phosphate binders, excess pill burden, and an inadequate understanding of a low phosphorus diet [7].

Appropriate, timely, and effective dietary interventions are recognised as an essential component to achieve a normal serum phosphate [8]. Unfortunately, dietary management of any kind in this cohort of patients also comes with the added challenge of limited and variable access to renal dietitians [9]. Studies have indicated that over 40% of renal dietitians lack adequate time with patients, and only one in four feel that they do not have appropriate technology and administrative support to implement evidence-based practice guidelines [10].

Given the rising disease prevalence, over-stretched health systems, and the complexity of managing the diet in patients receiving dialysis, there is a need for novel interventions that can not only help patients navigate daily challenges, but that could also be integrated into clinical practice to support the work of dietitians and provide more information without the need for face-to-face appointments. 

Nutrition education via a smartphone application (App) has been shown to be effective in improving outcomes in patients with diabetes, weight management, and smoking [11]. In renal nutritional intervention, several studies have shown improvements in sodium, potassium, and fluid intakes [12,13,14].

In this study, we aim to explore the perspectives of consumers regarding how they receive diet and lifestyle recommendations, the challenges they face in adopting them, and the acceptability of telehealth intervention to potentially support and improve their dietary self-management. Utilising consumer involvement, we also aim to consider how to incorporate this technology into a typical therapeutic regime that could help to improve the management of serum phosphate.

## 2. Materials and Methods

### 2.1. Participant Selection

Participants receiving maintenance dialysis (both haemodialysis (HD) and peritoneal dialysis (PD)) and caregivers within one of three West Australian metropolitan dialysis units were purposively sampled to achieve demographic diversity, including age, gender, socio-economic status, and clinical characteristics (e.g., time since diagnosis). All patients receiving dialysis at the unit were invited to attend through advertisements (posters/flyers) in the clinic reception area, via nephrologist outpatient clinics, and engagement with the lead investigators. Ethics approval for the above project was granted in accordance with the requirements of the National Statement on Ethical Conduct in Human Research (National Statement), and the policies and procedures of The University of Western Australia.

Eligible participants were adults aged 18 years or over, on dialysis for at least 3 months, have access to a mobile phone, and English speaking. Patients with known cognitive impairment that prevented them from providing informed consent and understanding the nature of the study were excluded. Of the 34 patients and caregivers registered for the workshops, 17 patients receiving dialysis (16 receiving HD and 1 receiving PD) and 5 caregivers actually consented and participated in the trial during the period between January and February in 2022. Those that did not attend either forgot, were unwell or had another appointment on the same day. We aimed for three focus groups to allow for failure to attend on the assigned day. Participation was voluntary and written informed consent was obtained from both patients and caregivers before the session commenced. Light refreshments were provided, and all participants received a USD 25 gift voucher for participating in the study.

### 2.2. Setting

Three focus groups lasting 60–90 min each were conducted in private meeting rooms in the satellite dialysis unit and research centre over a 2 month period in 2022. They were facilitated by the lead investigator (JB) and another investigator (CK) who recorded field notes and assisted with consent forms. Discussions developed from the semi-structured questions (Table 1) during the focus groups were audio recorded on a mobile phone App (Voice Memos) and later transcribed (otter.ai inc).

### 2.3. Data Collection

**Figure 1:**
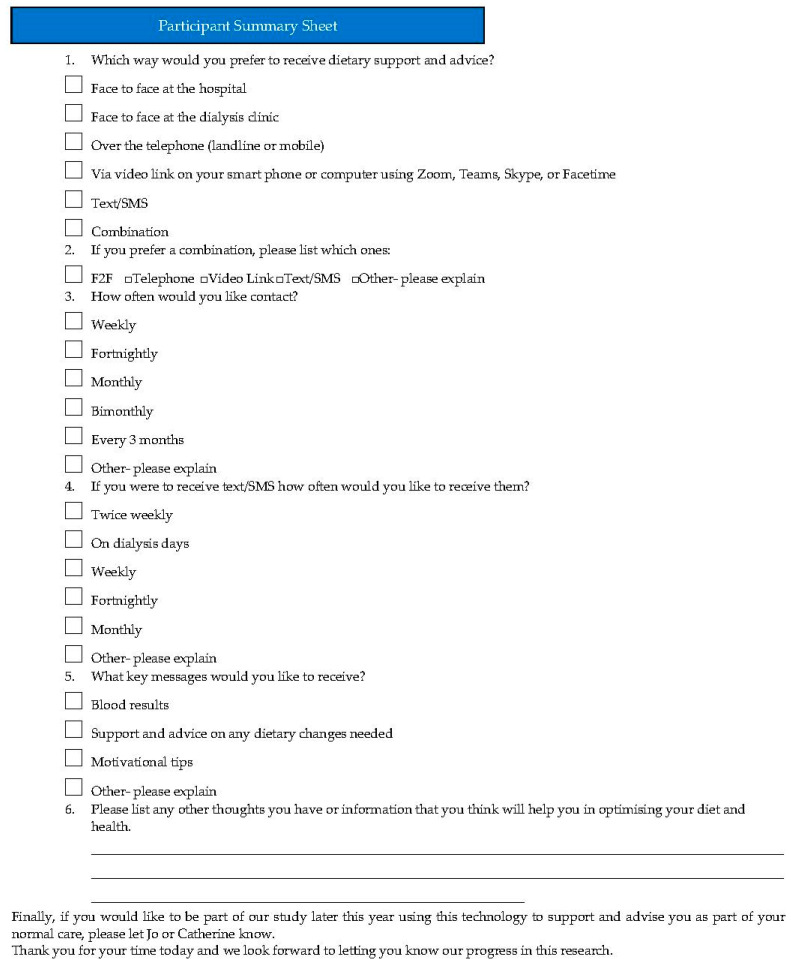
Participant summary sheet.

During each group, a variety of questions and vignettes were used to explore patient experiences (see Table 1). The questioning framework included feasibility, acceptability, technology barriers, dietary challenges, and preferred content of telehealth delivery methods. The questions were raised with the group as open questions with probing questions listed to assist the facilitator in directing the group and encouraging participation. Finally, a summary sheet (similar to the nominal group technique) was used at the end of the session for the participants to reflect on the discussions and provide their preferred method for mobile health package delivery (Figure 1). Theoretical saturation was agreed by the authors to have occurred by the third group. This is consistent with the literature which indicates that three focus groups were enough to identify all of the most prevalent themes within the data set [15].

### 2.4. Data Analysis—Theoretical Framework

This qualitative study utilised focus groups to elicit the perspectives of consumers regarding digital health and nutritional care. We assumed a relativist ontological position and utilised principles of grounded theory to undertake inductive thematic analysis. Grounded theory was used due to its ability to unravel the meaning of people’s interactions, social actions, and experiences in the area of interest.

Once transcribed, three investigators (JB, CK, and KL) independently reviewed the transcript line by line to complete the process of coding the data and develop the coding tree. Printed transcripts yielded key quotes, which were meticulously extracted and categorised on separate pieces of butcher’s paper depending on the meaning. Through iterative theme clustering, data saturation was achieved, leading to further refinement and clear thematic definitions. This was reviewed by the investigators and final themes and supporting quotations (data extracts) were collated. Transcribed data did not contain identifiable information. A summary of the preliminary findings was sent to the participants in a newsletter format to ensure that the analysis reflects the full range and depth of the data collected. This study is reported using the Consolidated Criteria for Reporting Qualitative Research Guidelines [16].

## 3. Results

Twenty-two participants (seventeen patients receiving dialysis, five caregivers), 47% male, mean age 71 (±standard deviation 13 years), participated in one of three focus groups (5–8 participants per group) between January and February 2022. Participant demographics are shown in Table 2.

Participants described a desire to learn more information about diet, but also described challenges to information. This overarching theme was described as “a desire to learn”. Four subthemes that facilitated learning were apparent and are illustrated in more detail in Table 3. Exemplary quotations are included in the following section which expands on these themes and clarifies these concepts. The quotations below are abbreviated to preserve participant anonymity and are cited as P (participant) focus group number.

**Theme** **1.** *Meaningful Communication*

*A need for improved communication about dietary advice*

Participants expressed frustration and confusion about the way dietary advice was presently communicated. Conflicting messages about diet between patients and health professionals have left consumers confused “I want more consistency (about diet information) from the team” (P group 1).

In addition, consumers desired positive messages about what they can eat and preferred not to focus on what they perceived should be avoided, e.g., “first time I saw a specialist he said keep away from bananas”(P group 3).

Tactful delivery of dietary advice was also desired. ”The young lady didn’t listen to me what I said, she just looked at my blood results and because my phosphate was high, she tore strips out of me. I walked out” (P group 2). Overall, they felt that current dietary advice lacked clarity and consistency.

**Theme** **2.** *Conducive Information*

*A preference for tailored, individualised, plain language dietary information*

Participants expressed the need for information to be provided in plain language, with preference for tailored and individualised advice. “A personalised approach I think is necessary as all kidney disease patients have different issues and the journey is everchanging, clearly diet is very important and deserves the focus that it needs” (P group 3). They reminded us that they were in a complex, demanding, and difficult situation; therefore, they needed clear dietary guidance.

The absence of these features was a barrier to behaviour change. “When I was given the (foods to avoid/foods to eat) list, I was literally scared to eat anything, and I lost weight, and it took a while to relax about it” (P group 2). Participants also reported feeling overwhelmed with the information they received. “At first I was bombarded with photocopied sheets—too many sheets of paper—quite overwhelming” and “We need something simpler” (P group 2).

**Theme** **3.** *Appropriate Timing*

*A desire for health advice at the right time*

Consumers described feeling overwhelmed with the volume of medical appointments and experienced difficulty attending face-to-face appointments in addition to dialysis treatments. They felt they were not supported enough “(I was) flung into the deep end at the beginning” (P group 2) and consumers expressed a desire for health advice to be provided in a timely manner. For example, regular reminders about treatments or appointments “regular reminders of what is good for us”, and access to advice following abnormal pathology results, “I want information every month when blood results come out from the dietitian and via SMS or phone would save me time and help me make changes” (P group 3). The timing of advice was also critical with consumers describing a desire for more intense education at the commencement of dialysis being balanced with feelings of being overwhelmed: “it comes as a sudden shock for most so don’t know what questions to ask”, “I think the first 6–12 months you need more help as it’s all so complicated” (P group 1), and “At the beginning I struggled as I got very confused, I was on so many drugs that I just didn’t remember any advice I was given” (P group 2).

**Theme** **4.** *Contemporary Modalities*

*Delivering information using different technologies*

When asked about the preferred mode of dietary advice to be delivered, consumers overwhelmingly preferred a combination of delivery methods with face-to-face delivery supplemented with text/SMS/App messages. They felt regular reminders via these delivery modes would facilitate learning and could be delivered in a timely manner. For example, “getting that (dietary) information in segments is a far better process than being hit with all the information in one go” (P group 1) and “An App is a good idea with alerts”(P group 2). Barriers to ongoing face-to-face care were described and included parking, waiting times, and costs. “I don’t mind going to an appointment now and again, but three times a week of four hours of dialysis is restricting and our costs so to get to other appointments is difficult” (P group 2) and “And you have to find parking, and then you sit there waiting, wasting your time, for me it’s a lot more convenient to be able to have a phone call or message” (P group 3).

Other consumers provided ideas about digital health strategies to engage consumers: “I like the idea of an App that can put results in for you and tells you what to cook or here is suggestion for this” (P group 2), “A type of monthly newsletter sent to my email or via an App”(P group 2), and “More visual information (images) and actually doing it” (P group 2). The preferred time period for focused ongoing education was described as at least three months after starting dialysis.

## 4. Discussion

This qualitative study aimed to investigate consumer perspectives regarding how they receive diet and lifestyle recommendations, the challenges they face in adopting them, and the acceptability of telehealth intervention to potentially support and improve their dietary self-management. Our participants reported a “desire to learn”, but felt that they were not supported enough to integrate this often difficult dietary plan with healthy eating guidelines due to the lack of timely, appropriate, and tailored information. They welcomed the use of telehealth to deliver regular nutritional care and support them in juggling the demands of their multifaceted dietary needs. Our participants felt strongly that the current information was confusing, not consistent between specialist health professionals, and untimely. This often left them exasperated, resulting in over restricting their intake and missing out on important key nutrients. 

Patients receiving maintenance dialysis and their caregivers also felt that the resources they were often given were outdated and not relevant to them; hence, the clear requirement for more individualised dietary information and advice that was positively and regularly delivered. The latter was a key problem due to time constraints not only for the patient but also the inadequate access to specialist renal dietitians, resulting in a reduced delivery of an essential service [17] and consumers often not ever being seen by a renal dietitian [18]. They especially felt that it was difficult if they were not only on dialysis but also had a diagnosis of diabetes, which is common in this population, with the information available being conflicting and complex. With this in mind, the use of novel approaches for delivering cost effective and timely education was welcomed by the participants.

Our findings confirm other qualitative literature that patients receiving dialysis often experience information overload, difficulty in adapting to restrictions, and lack dietary advice [19]. The Canadian Frailty Observations and Interventions Trial (canFIT) confirmed that the timing and delivery of dietary care were not appropriate and a better understanding of patients’ daily experiences and struggles is needed to deal with dietary recommendations [19]. 

In semi-structured interviews conducted with patients receiving maintenance dialysis, Stevenson et al. [20] found that patients reported fragmented, contradicting, and irrelevant advice which hindered their adherence to dietary recommendations. The authors concluded that the use of technology, such as mobile Apps or text messaging, could be considered to support self-management and education.

A meta-synthesis study of 23 qualitative studies confirmed patients’ perspectives that diet and fluid restrictions are a complex and challenging process involving a constant struggle, and that they are not supported enough [21]. In light of these findings, the authors recommended individual counselling services for diet and fluids to be increased.

A focus group study by Kelly et al. [22] with non-dialysis CKD patients considered their perspectives on telehealth and dietary management. They concluded that this had the potential to overcome shortcomings in current health service delivery and offer patients comprehensive and consistent information, which helps to encourage self-monitoring and management. Other studies considering patients’ readiness, interest, and capability in using mobile health concluded that this could provide an important alternative and/or supplemental resource for patients and families [23,24,25].

Several studies, such as KIDNEYTEXT [26] and ENTICE-CKD [27], as well as interventions by Kelly et al. [22] have explored the use of targeted text messaging services to facilitate increased frequency and quality of contact between the patient and health care professional in both CKD patients and those receiving dialysis. These pilot studies indicated that this form of intervention was both feasible and acceptable to patients to improve dietary behaviour patterns through goal setting and self-management. A recent systematic review by Kelly et al. [28] concluded that telehealth-delivered nutrition interventions appear to be a cost-effective option, in particular, mHealth modalities. Furthermore, this mode of education delivery can be cost effective compared with traditional care [29,30]. In agreement with the existing literature, we demonstrated that our participants felt that novel approaches (like mHealth) are an appropriate method to deliver information.

Patients’ experiences and perspectives are extremely valuable for informing the development of programs to overcome patient challenges and incorporate evidence-based diet and lifestyle information in a way that is both meaningful and will result in a higher acceptance to the program being proposed. Furthermore, design features that increase patient usability, optimise efficacy, and are supported by theories of behaviour change, are important to consider at inception [31]. Whilst digital nutritional counselling appears to be promising and with mobile phone ownership being high (84%) in patients with CKD, patient implementation and evaluation are stressed as being part of the development process [32].

More recently, emerging evidence is present in the current literature on digital health (smart phone applications, web based/software programs, and wearable devices) and the opportunity to use these modalities in self-management to enhance the nutritional care of people with CKD [26]. 

The strength of this study was shown in contrast to most qualitative studies that use surveys, we used focus groups that enabled us to achieve rich data on this topic. We also expanded on this by incorporating nominal group techniques to make the process simple and transparent, as well as encouraging contributions from everyone.

While the study involved enough participants to reach a theoretical saturation, we acknowledge that they may not fully represent the broader dialysis population. Factors like location (outside metro areas), timing constraints, language barriers, and age groups could limit inclusivity. To address this in future research, we will consider involving interpreters and, where relevant, encouraging more participants to bring their caregivers, who often play a key role in shopping and meal planning. Unfortunately, this was not possible in this study. Therefore, we are committed to learning from this experience and expanding our reach in future research.

## 5. Conclusions

Consumers highlighted the ongoing lack of dietary education to assist with behaviour change, confirming the current understanding from the literature. Emphasis was placed on appropriate nutrition intervention in alignment with the priorities of the patients such as time commitments and positive themes. Telehealth options would seem to be an acceptable mode to deliver nutritional care and dietary advice in a timely manner. With patient engagement being a focus for any digital intervention, this focus group will inform the design of future clinical trials to incorporate the use of telehealth to improve the management of serum phosphate.

## Figures and Tables

**Table 1:** Focus group semi-structured interview questions.

Welcome and Introduction + Ice Breaker We want to hear your thoughts and experiences on how you manage the dietary recommendations in relation to your kidney and dialysis treatment. What types of advice/coaching have helped you make changes, what motivates you to stay on track with dietary advice, what are some of the challenges you face, and what do you think about telehealth as an option for more regular coaching. All information discussed today is confidential. To assist us in putting together a summary of your responses, the session will be recorded; however, it will be deidentified to protect your privacy.
1. To start, chat with the person next to you and find out what has been your experience with nutritional care in relation to your kidney failure? List 1 positive and 1 negative outcome from that experience.
2. Who/what has helped you in the past with dietary advice and making positive changes? For instance, do you routinely use the internet or diet pamphlets?
3. How do you use technology, e.g., mobile phone, internet, skype, zoom?
4. What do you think about more regular appointments with the dietitian for advice and support to help with changes in your diet?
5. To improve delivery of dietetic advice/support, we are considering a combination of ways to achieve this, e.g., phone, email, text—has anyone had experience with this before?
6. If you were to receive additional dietary support via telehealth, when do you think it would be good to receive it?
7. Would you like more information about your kidney health, e.g., blood results?
8. What type of telehealth would you prefer—SMS or email?
9. If we were to SMS you, how often would you like to receive dietary input? Every other day, weekly, fortnightly, monthly? Would you prefer to receive information on a dialysis day or other day? What type of information would be most beneficial?
10. Let us summarise the responses using your participant summary sheet.

**Table 2:** Baseline demographics of the study cohort.

Characteristic	Number (%)
**Gender**	
Male	8 (47%)
Female	9 (53%)
**Age (years)**	
50–60	5 (29.4%)
61–70	4 (23.5%)
71–80	1 (5.9%)
80+	7 (41.2%)
Mean Age	70.7 (SD 13.4, Median 69, IQR 26)
**Ethnicity**	
White	15 (88.2%)
Asian	1 (5.9%)
Southeast Asia	1 (5.9%)
**Vintage (Mean in years)**	4.02 (SD 3.52, Median 3, IQR 4)
**Mode**	
Satellite centre haemodialysis	13 (76.5%)
Home haemodialysis	3 (17.6%)
Peritoneal dialysis	1 (5.9%)
**Education**	
Up to 10th Grade	3 (17.6%)
Up to 12th Grade	10 (58.8%)
Tertiary Education	4 (23.6%)
**Employment**	
Employed	6 (35.3%)
Retired	11 (64.7%)

**Table 3:** Illustrative quotes.

Theme	Illustrative Quotes
Meaningful Communication	“But it’s not that we are confused, you guys have confused us” (P group 2). “We want to be helped, we’re only human and we make mistakes, but we don’t want to be told off for it—that doesn’t help us”(P group 3). “She looked at me and said—well you won’t be able to eat those anymore. I thought that was a bit abrupt…” (P group 2). “And there’s a lot of misinformation about some foods, I am supposed to eat white bread, but then I need brown bread, and that’s where it’s confusing” (P group 1). “It’s very confusing one food item in one category is don’t eat it because you’ve got this, then in another category that same food you can eat…so its complicated” (P group 1). “But then if you speak to kidney specialists they will never talk to you about food—as far as they are concerned, food has nothing to do with anything” (P group 2).
Conducive Information	“ I was literally frightened to eat”( P group 2). “Clear reminders of where you can get information from e.g., renal dietitian, GP, dialysis unit/nurse” (P group 1). “I would like to feel that the information is tailored to me, my diagnosis, my lifestyle, my medication” (P group 3). “She gave me a whole wad of photocopied things from different places” (P group 2). “I agree with the reams and reams of paper…it makes it very impersonal” (P group 2). “I was bombarded with paper—too many loose pieces and no index like a book—didn’t make sense…” (P group 2).
Appropriate Timing	“I want more information at the beginning” (P group 2). “Yes, the first 3–6 months more information, more intense” (P group 1). “ Regular information just to keep yourself refreshed” (P group 3). “ But to see a dietitian regularly whether it be on the phone or zoom so you’re not having to go into the hospital, just so you can touch base with somebody cause you are constantly coming up with stuff and your journey changes as your kidney changes and other stuff happens” (P group 3). “I think one step at a time and getting information in segments is probably going to be a far better process than you’ve been hit with all the information in one go. It’s just overwhelming” (P group 3).
Contemporary Modalities	‘’Yeh telehealth, particularly during Covid, now find it very useful” (P group 1). “Telephone consultations with dietitians in your home—can be more informative…” (P group 2). “More visual information on what’s at the supermarket and going there then to look at it” P group 3). “Something like the liver App we use would be really helpful” (P group 2). “An App with alerts going to an inbox within the App” (P group 3). “I want something weekly via text/App especially when things change” (P group 2). “Reminders of what is good for us, new products on the market, top tips via text/phone messages” (P group 3). ‘’Regular support and motivational tips via text/App, face to face least choice as time and travel constraints” (P group 2). “A sort of repetitive reminder as short term memory problems, so that would be fantastic” (P group 1). “I want information every month when the blood results come out from dietitian via SMS/phone/App—it would save time and help me make change” (P group 2).

## Data Availability

The data presented in this study are available on request from the corresponding author.
